# Cadmium-induced endothelial dysfunction mediated by asymmetric dimethylarginine

**DOI:** 10.1007/s11356-020-08116-5

**Published:** 2020-03-02

**Authors:** Hamda A. Al-Naemi, Sandra Concepcion Das

**Affiliations:** 1 Laboratory Animal Research Center, Qatar University, P.O. Box 2713, Doha, Qatar; 2 Department of Biological and Environmental Sciences, College of Arts & Sciences, Qatar University, Doha, Qatar

**Keywords:** Endothelial nitric oxide synthase (eNOS), Asymmetric dimethylarginine (ADMA), Cadmium, Endothelial dysfunction

## Abstract

Cadmium (Cd) is a naturally occurring toxic heavy metal with no known essential biological functions. Exposure to Cd increases the risk of cardiovascular disease by disrupting vascular homeostasis at the endothelium. The aim of the study was to evaluate the effect of chronic low-dose Cd on vascular structure and function. Fifty adult male Sprague Dawley rats were grouped and assigned to one of two treatments for 14 weeks. The control group received normal water for 14 weeks while the Cd-treated group received 15 mg Cd/kg B.W. as CdCl_2_ in water for 10 weeks. A subset of the Cd-treated group received 15 mg Cd/kg B.W. as CdCl_2_ in water for 10 weeks followed by 4 weeks of normal water. Results show an overall decline in vascular function and structure. Withdrawal of Cd treatment showed a considerable restoration of vascular structure and vasorelaxation function. Additionally, asymmetric dimethylarginine (ADMA) bioavailability was found to be lowered over time. Interestingly, the expression of eNOS in the Cd-treated group was found to be significantly elevated during the exposure by more than 3-fold in comparison with that in the control group. This protein expression was similar to the control group after the withdrawal of Cd treatment. Taken together, the results suggest that ADMA, an eNOS inhibitor, may play a role in altering endothelial function in the presence of cadmium. In conclusion, the findings indicate that even at low doses, Cd leads to endothelial dysfunction mediated by ADMA.

## Introduction

Cadmium is a widely distributed environmental and industrial pollutant that has a negative impact on human health following long- and short-term exposure. Cadmium is released into the environment via anthropogenic activities like metal smelting, mining, fuel combustion, and use of phosphate fertilizers (Cullen and Maldonado 2013). Furthermore, it has been well established that cadmium is one of the considerable toxicants in tobacco smoke (Satarug and Moore 2004; Milnerowicz et al. 2015).

Human exposure to cadmium primarily occurs via ingestion of contaminated food and water, or inhalation. Studies have shown that cadmium causes organ damage by accumulation in various organs such as the liver, kidneys, lungs, testis, heart, bone, eye, and brain (Thévenod and Lee 2013). Current research in human and animal models shows that the vascular wall is also a target of cadmium deposition disrupting vascular homeostasis (Abu-Hayyeh et al. 2001; Prozialeck et al. 2008). Human population studies show inconsistent results. Chronic cadmium exposure in human subjects has been shown to induce endothelial impairment due to decreased nitric oxide bioavailability and increased asymmetric dimethylarginine (ADMA) (Lukkhananan et al. 2015). Furthermore, human exposure to cadmium has been associated with hypertension and high cadmium body burden (Franceschini et al. 2017; Garner and Levallois 2017; An et al. 2017). However, studies have also shown that there is no relationship between high blood pressure and cadmium body burden, with results varying by type of biomonitoring sample, sex, and smoking status (Tellez-Plaza et al. 2008; Agarwal et al. 2011; Lee et al. 2016).

In vivo exposure to cadmium in drinking water resulted in the attenuation of endothelial nitric oxide synthase (eNOS) protein expression in aorta of Sprague Dawley rats (Yoopan et al. 2008). It has been previously demonstrated that ingestion of 15 ppm of cadmium salt in drinking water intoxicated rats and showed serum cadmium concentrations up to the World Health Organization toxic limit (Larregle et al. 2008; Calderoni et al. 2010). Subchronic exposure to cadmium-contaminated water for 3 months resulted in an increase of systolic blood pressure and reduced acetylcholine-induced vascular response (Yoopan et al. 2006). Proposed mechanism of these changes includes the disruption of calcium channels and nitric oxide bioavailability by induction of oxidative stress inhibiting eNOS (Martynowicz et al. 2004; Cannino et al. 2009).

Even though the aorta is affected by cadmium at low concentrations below reference values, the mechanisms by which vascular homeostasis is disrupted is not clearly understood. There is a lack of reports showing the vascular effects of cadmium at a chronic low dose. In this study, we aim to evaluate the effect of vascular wall damage under a chronic dose of cadmium via ingestion to evaluate (1) the structural alterations in the aorta, (2) the vasoreactivities, and (3) its relation to ADMA bioavailability, to explain some of the mechanisms behind the cadmium-induced endothelial dysfunction.

## Materials and methods

### Study design and experimental procedures

Fifty adult (8-week-old) male Sprague Dawley (SD) rats were obtained from the Laboratory Animal Research Center (LARC), Qatar University, were housed in individually ventilated cages (IVC), and were maintained in standard conditions of room temperature (18–22 °C), relative humidity (40–60%), and on a 12-h light:12-h dark cycle. The rats were randomly assigned to one of two groups for 14 weeks: control group or Cd treatment (Cd-treated). The control group received drinking water for 14 weeks. The Cd-treated group received 15 mg Cd/kg B.W. as CdCl_2_ (BDH Chemicals, England) in water for 10 weeks followed by 4 weeks of drinking water. The animals were provided ad libitum access to standard rodent chow and drinking water during the experiment. Animals were sacrificed 10 weeks after cadmium treatment and at 4 weeks as recovery period after cessation of cadmium treatment under anesthesia with sodium thiopentone (40 mg/kg B.W., i.p.; Ilium, Australia), and fasting blood was collected from the retro-orbital sinus. Following blood collection, animals were euthanized. The thoracic aorta was dissected and divided into three parts: the first part was for vasoreactivity examination; the second part was fixed in 10% buffered formalin for histology analysis; and the third part was frozen in liquid nitrogen and preserved in − 80 °C for protein analysis.

All experimental procedures were approved by the Institutional Animal Care and Use Committee of Qatar University (Approval No. QU-IACUC 038/2017).

### Blood pressure measurement

Blood pressure was measured at different time points during the study at weeks 0, 5, 10, and 14. Animals were placed in a pre-warmed cage for 5 min and restrained during the recording protocol. Blood pressure was recorded by the non-invasive tail cuff method (CODA, Kent Scientific Corporation) for 10 cycles with 5 acclimatization cycles as previously described (Wang et al. 2017). The average of accepted cycles was calculated and analyzed.

### Vascular contractility analysis

Vascular contractility was studied by modifying a protocol described previously (Owu et al. 2013). Briefly, the isolated thoracic aorta was cleaned of connective tissues and segmented to approximately 1 mm in length. Free of connective tissue, the rings were mounted in a tissue chamber containing physiological salt solution with a composition (in mM) of the following: 112 NaCl, 5 KCl, 25 NaHCO_3_, 1.8 CaCl_2_, 0.5 KH_2_PO_4_, 1 MgCl_2_, 0.5 NaH_2_PO_4_, 10 glucose, gassed with 95% O_2_ and 5% CO_2_, maintained at a resting tension of 1.2 g at 37 °C. One end of the aorta was fixed while the other was mounted to a force transducer for isometric tension. The tension was measured by digital force output software (750TOBS, DMT). The rings were equilibrated for 15 min at 1.2 g. To analyze the effect of Cd treatment on contractile response, concentration–effect curves (10^−7^ to 10^−2^ M) were obtained for the agonist noradrenaline (NA) and for the vasodilator acetylcholine (Ach). Vasoconstriction response to NA is expressed as grams of tension. Vasodilator concentration–effect curves were obtained for rings pre-contracted at approximately 80% of maximal contraction. Relaxation induced by ACh was expressed as a percentage of NA-induced contraction.

### ADMA bioavailability analysis

ADMA levels in plasma were determined by using the ELISA method (DLD Diagnostika, Germany) as per the user manual described previously (Schulze et al. 2004). Aliquoted plasma was thawed and pre-treated to be acylated in a reaction plate. The acylated ADMA was detected using rabbit anti-*N*-acyl-ADMA antiserum with the enzyme conjugate anti-rabbit-immunoglobulin G peroxidase. The color reaction was stopped using a stopping solution and the optical density was read at 450 nm using a microtiter plate.

### Histological analysis

Dissected thoracic aortas were fixed in 10% buffered formalin for 24 h. The fixed tissues were dehydrated in alcohol series, infiltrated with xylene, and embedded in paraffin. The embedded tissues were sectioned at 5 μm and stained in hematoxylin and eosin (Xu et al. 2015). The stained sections were evaluated for general histopathological changes. Imaging was done using a microscope (Zeiss Primo Star) fitted with a camera (Canon Powershot A650).

### Protein expression analysis

Liquid nitrogen–frozen sections were mechanically pulverized, homogenized, and incubated for 30 min in cell lysis buffer (Thermo Scientific, USA) containing protease inhibitor cocktail (Thermo Scientific, USA). The homogenized samples were centrifuged at 10,000 rpm for 10 min at 4 °C. The supernatants were collected and quantified for protein concentrations by Bradford reagent (Bio-Rad, USA).

Immunoblotting protocol was adopted from a previously published study (Zhang et al. 2018) with modifications. Briefly, the protein samples (40 μg) were loaded and subjected to 6–10% SDS-polyacrylamide gels and blotted to polyvinylidene difluoride (PVDF) membranes (GE Healthcare Limited, Buckinghamshire). The membranes were washed with TBST (20 mM Tris-buffered saline and 0.1% Tween-20) for 5 min and incubated in a blocking buffer (3% skimmed dry milk in TBST) for 1 h with agitation. The membrane was then washed in TBST and incubated with specific primary antibody (Abcam), eNOS (ab76198, 1:750) and α-actin (ab5694, 1:100) at 4 °C overnight. The blots were washed and incubated with HRP-conjugated secondary antibody (Abcam, ab205719, 1:5000; ab6721, 1:2000). Immunoreactive bands were visualized using enhanced chemiluminescence (Thermo Scientific, USA).

For densitometric analysis, an image analysis system (Image Studio Lite Ver 5.2) was used to quantify the band intensities of eNOS and α-actin. The results were normalized to the expression of α-actin in each group and represented as a fold change.

### Statistical analysis

Data were expressed as mean ± S.E.M. Two-way analysis of variance (ANOVA) was used to determine statistical significance (GraphPad Prism 8.0). *P* < 0.05 was considered statistically significant.

## Results

### Effect of cadmium exposure on vasocontractility

**Fig. 1:**
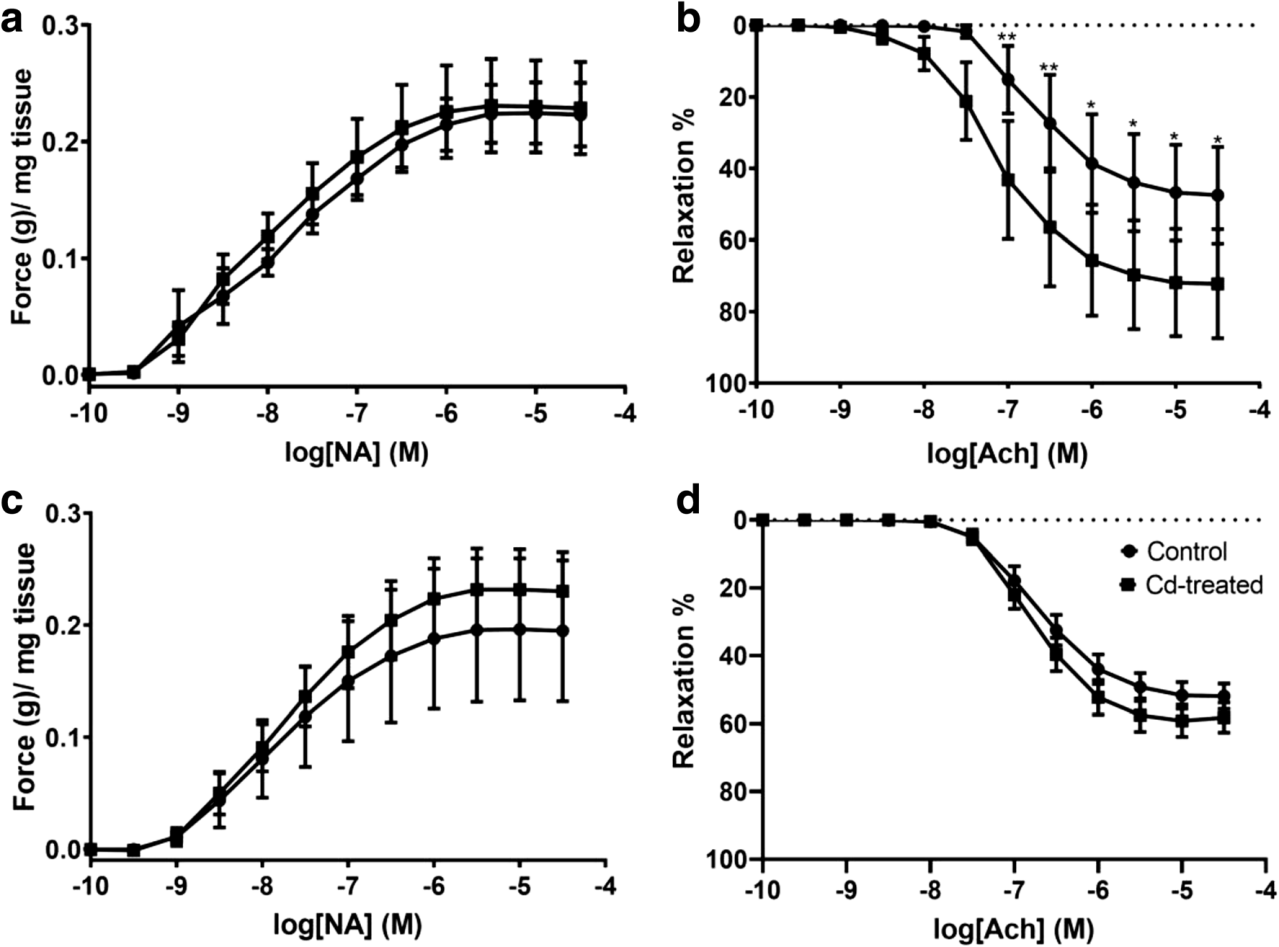
The effects of cadmium exposure on vasocontractility response of thoracic aorta of adult male SD rats to noradrenaline and acetylcholine. **a** Noradrenaline dose–response curve at week 10. **b** Acetylcholine dose–response curve at week 10. **c** Noradrenaline dose–response curve at week 14. **d** Acetylcholine dose–response curve at week 14. Data expressed as mean ± S.E.M. *P < 0.05, **P < 0.01

Vasoresponse to noradrenaline and acetylcholine was measured to assess the effect of cadmium exposure on contractility. No significant difference in the vasocontractility of the aorta at week 10 (Fig. 1a). Cadmium exposure followed by withdrawal increased the maximal contraction induced by noradrenaline (Fig. 1c). However, this increase was not statistically significant. The vasoresponse to acetylcholine is altered at week 10 (Fig. 1b). The Cd-treated group shows a statistically significant response beginning from 10^−7^ until 10^−4^ M. In Fig. 1d, the vasorelaxation response was unaffected.

### Effect of cadmium exposure on blood pressure

**Fig. 2:**
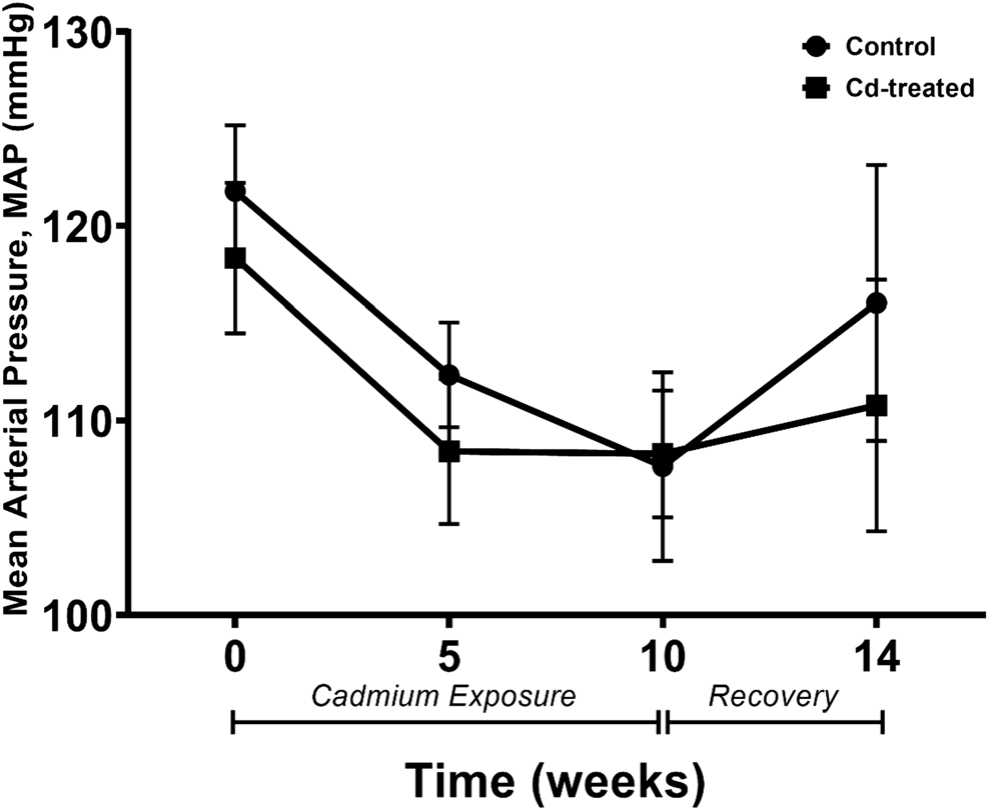
The effects of chronic cadmium exposure on blood pressure in adult male SD rats. Changes in mean arterial pressure over 14 weeks. Data expressed as mean ± S.E.M.

Mean arterial pressure was monitored at different time points and recorded. The results obtained show a steep decline in mean arterial pressure for 5 weeks followed by a steady increase until week 14 in the Cd-treated group (as shown in Fig. 2). This shift was not statistically significant against the control group.

### Effect of cadmium exposure on ADMA bioavailability

**Fig. 3:**
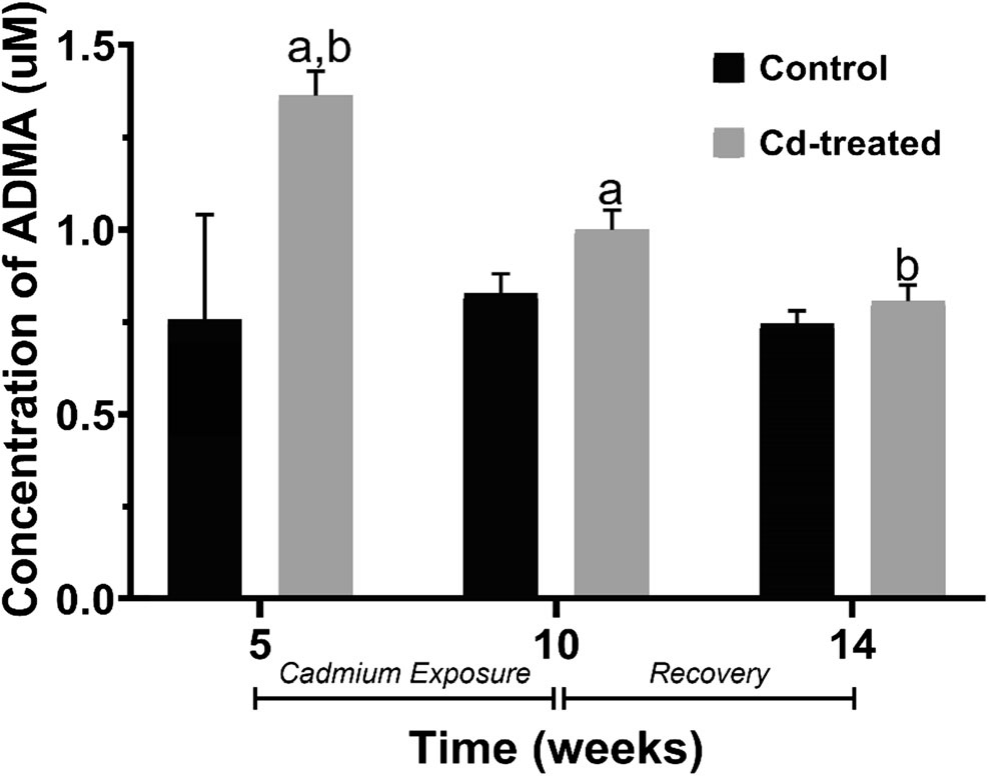
The effects of chronic cadmium exposure on bioavailability of ADMA in blood plasma of adult male SD rats. Data expressed as mean ± S.E.M. Columns sharing the same alphabet are statistically significant. a: *P* < 0.05, b: *P* < 0.05

Evaluation of the inhibitor ADMA bioavailability showed the highest bioavailable concentration at week 5 and gradually decreased until week 14 (Fig. 3). The difference of bioavailable concentrations from week 5 to week 10 and week 14 was found to be statistically significant (*P* < 0.05). When compared with the respective control group, ADMA increased by 80%, 21%, and 8% at week 5, week 10, and week 14, respectively. Overall, ADMA bioavailability increased by 35% compared with the control group.

### Effect of cadmium exposure on histological structure

**Fig. 4:**
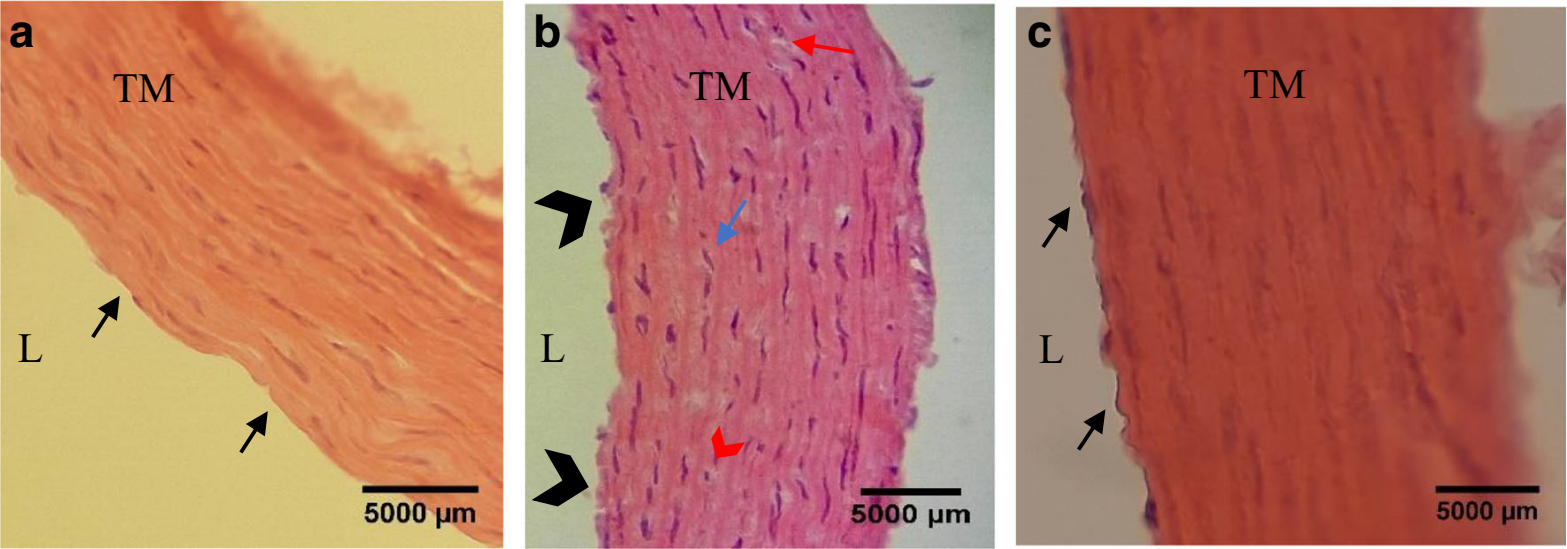
The effects of chronic cadmium exposure on aortic integrity of adult male SD rats. L indicates the luminal side showing aortic histological structure of the **a** control group with black arrows indicating a smooth, flat, and continuous endothelial layer of the TI and regular SMC arrangement in the TM; **b** Cd-treated group at week 10 with black arrow heads indicating denuded, discontinuous endothelium with irregular SMC in TM. Red arrow indicates formation of vacuoles, blue arrow indicates the hyperplasia of the SMC, and red arrow head indicates degeneration of the SMC; and **c** Cd-treated group at week 14 with black arrow indicating a continuous endothelial layer of the TI and regular organization of the SMC in the TM. (H & E, × 400)

To assess aortic integrity, hematoxylin–eosin-stained aortic sections were evaluated for alterations (Fig. 4). The control group showed a normal histological structure starting with a smooth, continuous endothelial cell (EC) layer in the *tunica intima* (TI) followed by a smooth muscle cell (SMC) layer in the *tunica media* (TM). The elastic fibers were arranged into continuous lamellae. At week 10, the Cd-treated group shows a rough EC layer with areas of denudation or disruption of continuity in the TI. Focal blood cell adhesions and subintimal thickening were also seen. There was an observed irregularity in the SMC arrangement of the TM accompanied with diffuse thickening. Irregularities were detected in the elastic laminae of the aorta wall (Fig. 4b). At week 14, the Cd-treated group showed a smooth, flat, and continuous arrangement of the endothelial cells in the TI accompanied with a regular smooth spindle-shaped arrangement of the SMC in the TM layer (Fig. 4c).

### Effect of cadmium exposure on eNOS expression

**Fig. 5:**
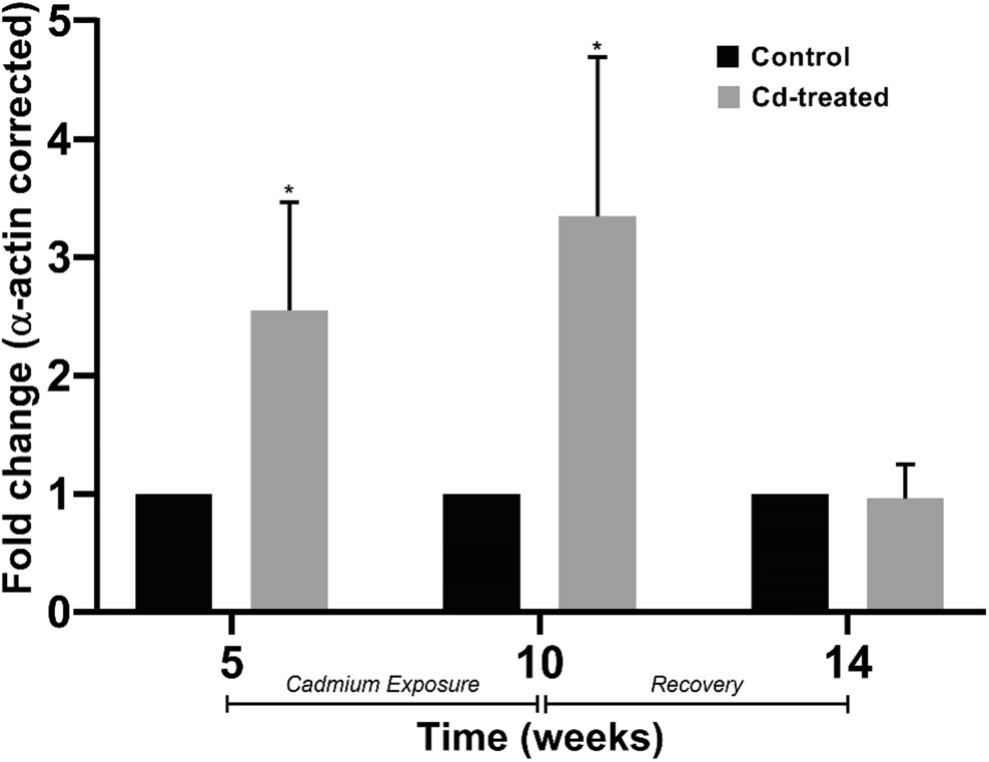
Effect of chronic cadmium exposure on eNOS expression. Fold change in eNOS expression in the aorta are represented above after correcting against α-actin. Data expressed as mean ± S.E.M, *n* = 4. *P < 0.05

Densitometric evaluation of the expression of eNOS shows an elevation in the expression of eNOS in the Cd-treated group (Fig. 5). In week 5, a 2.5-fold increase in eNOS expression was observed in the Cd-treated group compared with the control group. More than a 3-fold increase was observed for the Cd-treated group in week 10 compared with the control group. However at week 14, the eNOS expression in the Cd-treated group was similar with that in the control group. At week 14, the expression of eNOS was not statistically significant in comparison with that in the control group.

## Discussion

Epidemiological studies evaluating the association between prevalence of hypertension and Cd exposure have reported inconsistent results. Some studies have reported a positive association between Cd exposure and hypertension in the population (Tellez-Plaza et al. 2008; Garner and Levallois 2017; Wang and Wei 2018). These outcomes are contrary to those reported to show a negative correlation between Cd exposure and elevated blood pressure (Staessen et al. 1984, 1991, 2000; Nawrot et al. 2008; Garner and Levallois 2017). Animal studies have shown the effect of Cd exposure to elevation of blood pressure, particularly systolic pressure (Kacar Kocak et al. 2009). However, hypertension is associated with alteration in vascular structure and function. In the present study, the effects of chronic Cd exposure on vascular structure and function were studied. Adult male SD rats were exposed to daily Cd dose of 15 ppm for 10 weeks followed by withdrawal for 4 weeks.

The histological examination of the structural integrity of the aorta shows numerous changes to the endothelial and medial layer which is indicative of altered function. During Cd exposure, an array of structural changes was observed including abnormal and disorganized endothelium, fragmented elastic lamellae, degeneration of SMCs, formation of vacuoles, and thickening of the medial layer. During the recovery period, the vascular structure seems to have restored to normal arrangement. Increasing reports have reviewed the importance of the integrity of endothelial structure for viable vascular function (Kolluru et al. 2006; Messner et al. 2009; Knoflach et al. 2011; Oliveira et al. 2011). Additionally, there is a growing volume of evidence implicating the endothelium as a primary target of Cd toxicity (Prozialeck et al. 2008; Nagarajan et al. 2013). The components of the medial layer of the aorta—the smooth muscle cell (SMC) and connective tissue like elastin and collagen—contribute to the elasticity of the aorta (Shirwany and Zou 2010). Our findings are in accordance with those observed by Pérez Díaz et al. (2013) reporting irregular arrangement of luminal layers and cytoplasmic alterations of the endothelial cell layer in aortas of adult male Wistar rats treated with 15 ppm Cd for 2 months. In vitro experiments on endothelial cells have shown that Cd concentrations at low doses, below the currently considered toxic level, lead to increased cell permeability due to cell death (Messner et al. 2009; Pérez Díaz et al. 2013). Sangartit et al. (2014) reported the thickening and stiffening of the aortic wall in Cd-exposed mice which were associated with alterations in the vascular composition. Similar outcomes are also reflected in the current study suggesting altered mechanical forces during exposure to Cd that may be triggered by adaptive restructuring of the aorta.

Besides structural changes, functional changes may superimpose to perturb vascular health, thereby altering the function of the aorta. This study has shown an overall depression in mean arterial pressure. The abrupt fall in mean arterial pressure between baseline (week 0) and week 5 may be attributed to the lack of conditioning of the animals to the blood pressure protocol. A review by Nomiyama and Nomiyama (2000) suggests that changes in blood pressure are dependent on the Cd dose such that prolonged exposure of a low dose may elevate blood pressure marginally whereas a higher Cd dose may depress blood pressure, only in hypertensive individuals. This suggests that Cd exposure tends to alter blood pressure in hypertensive animals. There is evidence to suggest that heart rate is unaltered by Cd exposure. A study by Ozturk et al. (2009) claimed that chronic Cd exposure at 15 ppm for 60 days has no effect on the heart rate of 3-month-old male Wistar albino rats. The current study used similar daily dose (15 ppm of CdCl_2_) for 10 weeks and showed no statistically significant alteration in the heart rate. Despite differences in the period of exposure, age, and strain of the animals used in the study, our findings are in agreement with the prior cited references.

An analysis of the effects of Cd exposure on contractile vasoresponse shows a statistically significant leftward shift in the Ach-induced relaxation after 10 weeks and a slight restoration of vasorelaxation response after withdrawal of Cd treatment. However, this restoration of vasorelaxation response was not statistically significant. Earlier studies reported a decrease in the Ach-induced vasorelaxation (Göçmen et al. 2000; Gökalp et al. 2009). Yoopan et al. (2008) predicted that Cd may interact with the thiol groups of muscarinic receptors altering the ligand binding site consequently decreasing the muscarinic receptor responses to Ach. Alternatively, Gökalp et al. (2009) observed a reduction in the endothelium-dependent relaxation attributed to Cd-induced inhibition of nitric oxide (NO) formation.

One of the factors implicated in the prognosis of cardiovascular disease is the bioavailability of NO in circulation. To elucidate whether the attenuation in Ach-induced vasorelaxation is mediated by the eNOS endogenous inhibitor—asymmetric dimethyl arginine (ADMA)—bioavailability was studied. The results of this study show a significant increase in ADMA after 5 weeks of Cd exposure that decreases over time until after withdrawal. This study supports evidence from clinical observations that subjects with prolonged heavy metal exposure exhibit endothelial dysfunction mediated by increased ADMA in circulation (Lukkhananan et al. 2015; Ochoa-Martínez et al. 2018).

A strong relationship between bioavailability of ADMA and NO has been reported in literature (Sibal et al. 2010; Osorio-Yáñez et al. 2017; Chen et al. 2018; Liu et al. 2018). One of the factors influencing the bioavailability of NO is eNOS expression. Results of eNOS expression show an upregulation in the expression. A study by Takahashi et al. (2004) reported a significant upregulation in the expression of eNOS along with a slight increase in Ach-induced relaxation in acutely Cd-treated aortic strips. The findings of our study are consistent with that of Takahashi et al., suggesting that the increase in Ach-induced relaxation through eNOS activation mediated by NO is associated with the elevated expression of eNOS in the Cd-treated group at week 10. This further corroborates the decrease in the Ach-induced relaxation in the aorta of Cd-treated rats at week 14 that follows the pattern of expression of eNOS. Takahashi et al. (2004) explained that the augmented eNOS expression by Cd administration, which may lead to increased suppression of vasoconstriction, could also augment contraction by α-adrenoreceptor activation.

Cadmium is known to induce oxidative stress by either overwhelmed production of reactive oxygen species (ROS) or weakening antioxidant defense mechanisms, or a combination of both (Cuypers et al. 2010). The eNOS expression in vitro in human coronary artery endothelial cells (HCAECs) grown in culture and in vivo in intact animals was upregulated by an increase in ROS activity (Zhen et al. 2008). This effect appears to be partially mediated by limiting the bioavailability of NO, thereby exerting a negative feedback on the expression of eNOS by activating a transcription factor, nuclear factor-kappa B (Zhen et al. 2008). This suggests that further experiments looking at the role of nuclear factor-kappa B on the internal inhibition of eNOS expression could help to understand the impact of Cd toxicity on the vascular system.

In overview, chronic Cd exposure alters vascular structure inciting adaptive mechanisms represented by altered vascular function. Interestingly, a 4-week recovery from the Cd treatment seems to improve vascular structure and function. The findings of this study further support the hypothesis that even at low doses, not only is the endothelium a primary target of Cd toxicity but the vascular SMC as well. Further studies aimed at the role of endogenous inhibitors (like ADMA) are needed to understand the pathway by which Cd mediates vascular function and structure.
